# Bufei Jianpi Formula Improves Mitochondrial Function and Suppresses Mitophagy in Skeletal Muscle via the Adenosine Monophosphate-Activated Protein Kinase Pathway in Chronic Obstructive Pulmonary Disease

**DOI:** 10.3389/fphar.2020.587176

**Published:** 2020-12-17

**Authors:** Jing Mao, Ya Li, Suxiang Feng, Xuefang Liu, Yange Tian, Qingqing Bian, Junzi Li, Yuanyuan Hu, Lanxi Zhang, Huige Ji, Suyun Li

**Affiliations:** ^1^ College of Pharmacy, Henan University of Chinese Medicine, Zhengzhou, China; ^2^ Henan Key Laboratory of Chinese Medicine for Respiratory Disease, Henan University of Chinese Medicine, Zhengzhou, China; ^3^ Institute for Respiratory Diseases, The First Affiliated Hospital, Henan University of Chinese Medicine, Zhengzhou, China; ^4^ Co-Construction Collaborative Innovation Center for Chinese Medicine and Respiratory Disease by Henan and Education Ministry of P.R. China, Henan University of Chinese Medicine, Zhengzhou, China

**Keywords:** mitophagy, mitochondrial biogenesis, chronic obstructive pulmonary disease, Bufei Jianpi formula, skeletal muscle dysfunction

## Abstract

Skeletal muscle dysfunction, a striking systemic comorbidity of chronic obstructive pulmonary disease (COPD), is associated with declines in activities of daily living, reductions in health status and prognosis, and increases in mortality. Bufei Jianpi formula (BJF), a traditional Chinese herbal formulation, has been shown to improve skeletal muscle tension and tolerance via inhibition of cellular apoptosis in COPD rat models. This study aimed to investigate the mechanisms by which BJF regulates the adenosine monophosphate-activated protein kinase (AMPK) pathway to improve mitochondrial function and to suppress mitophagy in skeletal muscle cells. Our study showed that BJF repaired lung function and ameliorated pathological impairment in rat lung and skeletal muscle tissues. BJF also improved mitochondrial function and reduced mitophagy via the AMPK signaling pathway in rat skeletal muscle tissue. *In vitro*, BJF significantly improved cigarette smoke extract-induced mitochondrial functional impairment in L6 skeletal muscle cells through effects on mitochondrial membrane potential, mitochondrial permeability transition pores, adenosine triphosphate production, and mitochondrial respiration. In addition, BJF led to upregulated expression of mitochondrial biogenesis markers, including AMPK-α, PGC-1α, and TFAM and downregulation of mitophagy markers, including LC3B, ULK1, PINK1, and Parkin, with increased expression of downstream markers of the AMPK pathway, including mTOR, PPARγ, and SIRT1. In conclusion, BJF significantly improved skeletal muscle and mitochondrial function in COPD rats and L6 cells by promoting mitochondrial biogenesis and suppressing mitophagy via the AMPK pathway. This study suggests that BJF may have therapeutic potential for prophylaxis and treatment of skeletal muscle dysfunction in patients with COPD.

## Introduction

Chronic obstructive pulmonary disease (COPD), a common and preventable disease, is characterized by persistent airflow limitations caused by chronic inflammatory responses to noxious particles and gases (Patel et al., 2019). In China, COPD has become the third leading cause of death and the third most prevalent chronic disease (Collaborators, 2015), with an estimated 99.9 million individuals aged 20 years or older with spirometry-defined COPD (Wang et al., 2018a). Skeletal muscle dysfunction (SMD), a serious systemic comorbidity of COPD (Ceco et al., 2017), has been recognized as an early disease feature and an important causative factor in the limited mobility and mortality of patients with this disease (Maltais et al., 2014; Barreiro, 2016). Studies have shown that muscle wasting is experienced in up to 30%–40% of COPD patients, with significant impairments in activities of daily living, health status, and prognosis (Mowery, 2017).

Development of SMD is likely multifactorial, with inflammation and mitochondrial dysfunction playing predominant roles. Other pathophysiological factors, including oxidative stress, imbalance of protein synthesis/degradation, hypoxia, and cellular apoptosis have also been postulated to contribute to the muscle wasting experienced by COPD patients (Barreiro and Gea, 2016). Recent studies have identified major mitochondrial abnormalities in COPD skeletal muscle cells, including decreases in mitochondrial density and biogenesis, reductions in oxidative capacity, impairments in activity and coupling of mitochondrial respiratory chain complexes, increases in production of mitochondrial reactive oxygen species (ROS), and enhancements in autophagy and apoptosis (Meyer et al., 2013; Taivassalo and Hussain, 2016).

Decreased mitochondrial biogenesis and enhanced mitophagy play particularly important roles in mitochondrial impairment. Peroxisome proliferator-activated receptor gamma coactivator (PGC)-1α has been shown to be a master regulator of mitochondrial biogenesis (Tadaishi et al., 2011). PGC-1α acts as a cotranscriptional regulatory factor that induces mitochondrial biogenesis by activating nuclear respiratory factors (NRF) 1 and 2, which then activate mitochondrial transcription factor A (TFAM), an essential factor for replication, maintenance, and transcription of mitochondrial DNA (Grabacka et al., 2016; Yu et al., 2017).

Mitophagy, on the other hand, is closely associated with the AMPK pathway. AMPK directly facilitates phosphorylation of UNC-51-like kinase (ULK1), which stimulates the mitophagic activity of ULK1 (Allavena et al., 2016). The PTEN-induced putative kinase 1 (PINK1)/Parkin pathway has also been implicated as an important molecular mechanism mediating mitophagy. While PINK1 is a protein kinase primarily located in the mitochondrial outer membrane, Parkin is an E3 ubiquitin ligase generally located in the cytoplasm. When mitochondrial damage occurs, PINK1 phosphorylates the Parkin ubiquitin ligase at S65 and initiates its recruitment to the mitochondria. Once activated, Parkin ubiquitinates substrates on the outer mitochondria for two divergent processes, autophagosome recruitment and proteasomal degradation of ubiquitinated mitochondrial substrates. Mitochondrial fission-1 protein (Fis1), a receptor on the outer membrane, governs the developing light chain (LC) 3 isolation membrane to generate an autophagosome around the damaged mitochondria. The autophagosome is then delivered to the lysosome for degradation (Hardie, 2011; Wei et al., 2017).

Traditional Chinese medicine (TCM) has been successfully used to treat COPD for thousands of years. Lung-spleen qi deficiency syndrome is believed to be the main underlying pathologic mechanism for the SMD observed in patients with COPD (Medicine, 2012). Bufei Jianpi formula (BJF), which is composed of 12 medicinal herbs, has been proven to have efficacy in patients with COPD by improving respiratory function, reducing the frequency of acute exacerbations, and enhancing exercise tolerance (Li et al., 2012a). In previous experimental studies, we also found that BJF significantly improved skeletal muscle tension and tolerance in COPD rats, reduced cellular apoptosis, and improved mitochondrial function (Dong et al., 2015; Mao et al., 2019). It remains unclear, however, if mitochondrial dysregulation underlies the SMD observed in COPD.

In this study, we aimed to explore the mechanism by which BJF affects mitochondrial regulation via the AMPK pathway in an *in vivo* COPD rat model and an *in vitro* cigarette smoke extract (CSE)-induced skeletal muscle cell line to identify potential underlying mechanisms and to provide a basis for further research.

## Materials and Methods

### Reagents and Animals

*Klebsiella pneumoniae* (strain: 46114) was obtained from the National Center for Medical Culture Collections (Beijing, China). HongqiQu® filter cigarettes (tobacco-type of tar: 10 mg, nicotine content: 1.0 mg, carbon monoxide fumes: 12 mg) were purchased from Henan Tobacco Industry (Zhengzhou, China). Aminophylline (APL) tablets were obtained from Shandong Xinhua Pharmaceutical Co., LTD. (lot H37020630) (Shandong, China). 5-Aminoimidazole-4-carboxamide ribonucleotide (AICAR) was purchased from Selleck Chemicals (lot 2627-69-2) (Houston, TX, USA). Dorsomorphin dihydrochloride (BML-275 dihydrochloride) was purchased from MedChemExpress (lot 1219168-18-9) (Shanghai, China).

Thirty-six male and 36 female Sprague-Dawley (SD) rats with an average body weight of 200 ± 20 g were purchased from the Experimental Animal Center of Henan Province (Zhengzhou, China). The rats were housed in specific pathogen-free cages under standard temperature (25 ± 2°C) and light conditions, with a 12-h light/dark cycle, and free access to food and water. All animal studies were conducted in accordance with the Institute’s Guide for the Care and Use of Laboratory Animals and were approved by the Experimental Animal Care and Ethics Committee of the First Affiliated Hospital of the Henan University of Chinese Medicine.

### Preparation of Herbal Medicines

BJF, consisting of 12 herbs (Table 1), was prepared by the Laboratory of Pharmacology in Henan University of Chinese Medicine. All herbs were prepared in a fluid extract in accordance with the standard operating procedures. The high performance liquid chromatography (HPLC) fingerprint was performed to identify the main chemical constituents in BYF. A highresolution HPLC column (Venusil XBP C18, 4.6 × 250 mm, 5 μm) (Thermo Scientific, United States) were used at a flow rate of 1.0 ml/min at 30°C. Mobile phase A was 0.1% formic acid in water and mobile phase B was acetonitrile. The gradient elution program was 5–30 min, 5–100% B. The UV spectrophotometer detector was set at 270 nm.

**TABLE 1:** Components of BJF.

Chinese name	Name for publishing	Amount (g)	Lot. No	Company
Dang Shen	Codonopsis pilosula (Franch.) Nannf.	15	19110102	Zhengzhou Ruilong Pharmaceutical Co. Ltd.
Huang Qi	Astragalus mongholicus Bunge	15	19070102	Zhengzhou Ruilong Pharmaceutical Co. Ltd.
Bai Zhu	Atractylodes macrocephala Koidz	12	19110202	Zhengzhou Ruilong Pharmaceutical Co. Ltd.
Fu Ling	Poria cocos (Schw.) Wolf	12	19100102	Zhengzhou Ruilong Pharmaceutical Co. Ltd.
Huang Jing	Polygonatum kingianum Collett & Hemsl	15	191001	Bozhou Guangyuan Tang Traditional Chinese Medicine Decoction pieces Co. Ltd.
Zhe Bei Mu	Fritillaria thunbergii Miq	9	19060101	Zhengzhou Ruilong Pharmaceutical Co. Ltd.
Di Long	Pheretima aspergillum (E. Perrier)	12	19100102	Zhengzhou Ruilong Pharmaceutical Co. Ltd.
Hou Po	Magnolia officinalis Rehder and E. H. Wilson	9	19100102	Zhengzhou Ruilong Pharmaceutical Co. Ltd.
Chen Pi	Citrus reticulata Blanco	9	19120103	Zhengzhou Ruilong Pharmaceutical Co. Ltd.
Zi Wan	Aster tataricus L.f.	9	CP-427-191101	Bozhou Shenglin Pharmaceutical Co. Ltd.
Ai Di Cha	Ardisia japonica (Thunb.) Blume	15	191201	Anhui Jishun Traditional Chinese Medicine Decoction pieces Co. Ltd.
Yin Yang Huo	Epimedium brevicomu Maxim	6	190201	Hebei Sirui Pharmaceutical Co. Ltd.

### Preparation of BJF-Containing Serum

Sixty Sprague-Dawley rats were randomized into control and Bufei Jianpi groups. Rats in the Bufei Jianpi group were administered BJF (4.5 1g/kg/d) intragastrically 10 times once per day for 7 days. Rats in control group were administrated distilled water (2 ml per animal). On day 7, all rats were narcotized with 4% chloral hydrate and blood samples were collected, centrifugated at 3,500 rpm for 15 min, inactivated at 56°C, and stored in a −80°C freezer for further study.

### Preparation of COPD Rat Model and Drug Administration

Seventy-two rats were randomly assigned to the following groups: control, model, Bufei Jianpi Formula (BJF), 5-Aminoimidazole-4-carboxamide ribonucleotide (AICAR), Bufei Jianpi Formula + 5-Aminoimidazole-4-carboxamide ribonucleotide (BJF + AICAR), and Aminophylline (APL), with 12 animals in each group. The detailed procedures for creation of the COPD rat model have been described in a previous report (Li et al., 2012b); however, in brief, rats were exposed to cigarette smoke (concentration: 3,000 ± 500 ppm) for 30 min twice per day from week 1 through week 12. In addition, 0.1 ml of *Klebsiella pneumonia* suspension (6 × 10^8^ CFU/mL, 100 μL) was slowly dropped into the nasal cavities of rats every 5 days for the first 8 weeks. The successful generation of a COPD rat model was evaluated based on symptoms, lung function, and pulmonary pathology (Li et al., 2012a).

Starting from week 9, rats in the control and model groups were intragastrically administered normal saline (2 mL/animal). BJF (4.51 g/kg/d) was administered to rats in the BJF and BJF + AICAR groups, while APL (2.7 mg/kg/d) was administered to rats in the APL group twice per day for 12 weeks. AICAR (25 mg/kg/d) was intraperitoneally administered to rats in the AICAR and BJF + AICAR groups once a day during week 20. The equivalent dosages of BJF and APL were calculated using the equation:

$$D_{rat}\;=\;D_{human}\;\times \;\left(\frac{I_{rat}}{I_{human}}\right)\times {\left(\frac{W_{rat}}{W_{human}}\right)}^{2/3}$$

in which *D* represents dose, *I* represents body shape index, and *W* represents body weight.

All rats underwent necropsy after intraperitoneal injection of 10% chloral hydrate at 100 mg/kg of body weight at the end of week 20, and lung and quadriceps muscle tissues were collected for further analysis.

### Respiratory Function Analysis in Rat Model

Lung function was measured by a whole-body plethysmography system (Buxco Inc., Wilmington, NC, USA) at weeks 0, 4, 8, 12, 16, and 20. Tidal volume (TV), peak expiratory flow (PEF), and 50% tidal volume expiratory flow (EF50) were evaluated.

### Lung and Skeletal Muscle Histopathology in Rat Model

After being fixed in 10% formalin for 72 h, left lung and quadriceps muscle tissues were embedded with paraffin, cut into 4-μm thick sections, and stained with hematoxylin and eosin (HE) for light microscopy.

### Skeletal Muscle Ultrastructure Morphology in Rat Model

After being cut into sections with 1-mm thicknesses, fresh quadriceps muscle tissue was fixed in 2.5% glutaraldehyde for 2 h followed by 1% osmium tetroxide. Sections were then stained with uranyl acetate, dehydrated in a methanol series and propylene oxide, and embedded with Epon-812. Ultrathin sections were obtained, and ultrastructural changes and mitophagy of skeletal muscle cells were examined using a JEM-1400 transmission electron microscope (OLYMPUS, Japan).

### Measurement of Mitochondrial Function in Skeletal Muscle

Mitochondrial characteristics, including mitochondrial membrane potentials (MMP), ATP production, and opening of mitochondrial permeability transition pores (MPTPs) were measured with a JC-1 kit (Beyotime Biotech Co., Ltd., Shanghai, China), an ATP assay kit (Beyotime Biotech Co., Ltd., Shanghai, China), and an MPTP assay (Genmed Scientifics Inc., DE, USA), respectively, according to the manufacturer’s instruction and as previously described (Mao et al., 2019).

### Cell Culture and Transfection

Rat L6 myoblasts (obtained from the Cell Bank of Chinese Academy of Sciences, Shanghai) were cultured in Dulbecco’s modified Eagle’s medium (DMEM) (Solarbio Tech Co., Ltd., Beijing, China) supplemented with 10% fetal bovine serum (FBS) in a 5% CO_2_-humidified atmosphere at 37°C. When cells had grown by 70–90%, they were plated on dishes at a density of 1 × 10^5^ cells/mL and incubated in 5% CO_2_-95% air for 24 h. On the next day, cells were treated with 10% cigarette smoke extract (CSE), 10% CSE **+** AICAR (2.5 ng/mL), or 10% CSE **+**10% BJF-containing serum in DMEM and harvested 24 h later.

### Preparation of Cigarette Smoke Extract

CSE was prepared using a modified version of Sheridan’s method (Sheridan et al., 2015). Briefly, a filterless HongqiQu cigarette was combusted through a modified 50-ml syringe apparatus, and the smoke was dissolved in 20 ml of DMEM. The pH was adjusted to 7.4 with 1 mol/L sodium hydroxide and 37.5%-concentrated hydrochloric acid, and then diluted with DMEM until the optical density was measured to be 2.0 ± 0.05 using an ultraviolet spectrophotometer (Thermo Fisher Scientific, MA, USA). This solution represented “100%” strength. Smoked medium was then passed through a 0.22-µm filter to sterilize the solution. Finally, smoked medium was diluted in DMEM to the required strength and used for the experiment within 30 min.

### Measurement of Mitochondrial Respiration in L6 Cells

Mitochondrial respiration was measured with a Seahorse XF Cell Mito Stress Test Kit (Agilent Technologies, CA, United States) on a Seahorse XF24 Extracellular Flux Analyzer (Seahorse Bioscience, MA, United States) according to the manufacturer’s instruction. L6 cells were seeded (10,000 cells/per well) onto Seahorse assay plates in DMEM media and treated with 10% CSE for 24 h. During the experiment, cells were switched to an XF assay medium (HCO3-free modified DMEM) supplemented with 10 mM of L-glutamine, 10 mM of glucose, and 10 mM of pyruvate, while a pH of 7.4 was maintained at 37 °C. After baseline measurement of oxygen combustion rate (OCR), a mitochondrial respiration test was performed by sequential additions of 1.0 μM oligomycin, 2.0 μM carbonyl cyanide 4-(trifluoromethoxy) phenylhydrazone, and 0.5 μM rotenone/antimycin A. Several mitochondrial parameters were determined, including basal respiration, maximal respiratory capacity, proton leak, and non-mitochondrial respiration. The results were expressed as OCRs (pmol/min/lg protein).

### Measurement of Mitochondrial Membrane Potentials in L6 Cells

For measurement of mitochondrial membrane potentials, L6 cells were seeded in 6-well plates with a density of 1 ×10^5^ cells per well and treated with 10% CSE, 10% CSE + AICAR, or 10% CSE +10% BJF-containing serum for 24 h. Before the experiment, cells were treated with carbonyl cyanide *m*-chlorophenyl hydrazone (CCCP) (100 μM, Sigma) for 1 h as a positive control. Changes in mitochondrial membrane potentials of L6 cells in different groups were then assessed using the JC-1 mitochondrial membrane potential detection kit according to the manufacturer's instruction. Red fluorescence represented JC-1 aggregates, whereas green fluorescence represented the monomeric form of JC-1. Fluorescence intensity was analyzed by a Varioskan® Flash Microplate Reader (Thermo Fisher Scientific, MA, USA). MMPs in each group were calculated as the ratio of red/green fluorescence. In addition, cells were visualized using confocal laser scanning microscopy at a 514-nm excitation and a 529-nm emission for green and a 585-nm excitation and a 590-nm emission for red.

### Measurement of ATP Levels in L6 Cells

For measurement of ATP levels, L6 cells were seeded in 6-well plates with a density of 1 ×10^5^ cells per well for 24 h. After treatment with study drugs, cells were lyzed using a cell lysis reagent, and the ATP content in cell lysates was measured using an ATP assay kit (Beyotime Biotech Co., Ltd., Shanghai, China) following the manufacturer’s protocol. The total and mitochondrial protein concentrations were determined using a bicinchoninic acid (BCA) protein assay kit (Beyotime Biotech Co., Ltd., Shanghai, China).

### RNA Isolation and Real-Time PCR Analysis

Expression levels of mRNA in quadriceps muscle tissuewere analyzed by semiquantitative polymerase chain reactions (qPCR). Total RNA was extracted from the quadriceps muscle using a total RNA isolation kit (Solarbio Tech Co., Ltd., Beijing, China) according to the manufacturer's protocol. Real-time (RT)-qPCR was performed using a reverse transcriptase kit (Solarbio Tech Co., Ltd., Beijing, China) and a SYBR Green Master Mix (Vazyme Biotech Co., Ltd. Nanjing, China). To normalize the amount of total RNA in each reaction, a reference gene (*GAPDH*) was used as an internal standard.

### Western Blotting

Total proteins from cells or tissue samples were lyzed in a lysis buffer and quantified using a BCA assay. Ten-cell lysates were loaded into each well in 10% sodium dodecyl sulfate polyacrylamide gel electrophoresis (SDS-PAGE) gels and transferred to a polyvinylidene difluoride (PVDF) membrane. Membranes were blocked in 5% nonfat milk in tris-buffered saline and polysorbate 20 (TBST) for 2 h at room temperature and incubated with the indicated primary antibodies, including anti-AMPK-α, anti-*p*-AMPK-α, anti-PGC-1α, anti-TFAM, anti-LC3B, anti-ULK1, anti-PINK1, anti-Parkin, anti-mammalian target of rapamycin (mTOR), anti-sirtuin 1 (SIRT1), anti-peroxisome proliferator-activated receptor (PPAR)γ (Abcam, Cambridge, United Kingdom), or anti-glyceraldehyde 3-phosphate dehydrogenase (GAPDH) (Proteintech, Wuhan, China) at 4°C overnight. After incubation with the HRP-coupled secondary antibody (Proteintech, Wuhan, China) at room temperature for 2 h, signals were detected using a super-enhanced chemiluminescence plus reagent and scanned and quantified by a ChemiDoc MP imaging system (Bio-Rad, CA, USA).

### Statistical Analysis

Data are expressed as mean ± SD. Statistical differences between the groups were identified by one-way analysis of variance (ANOVA). Differences were considered to be significant at *P*-values of < 0.05.

## Results

**FIGURE 1:**
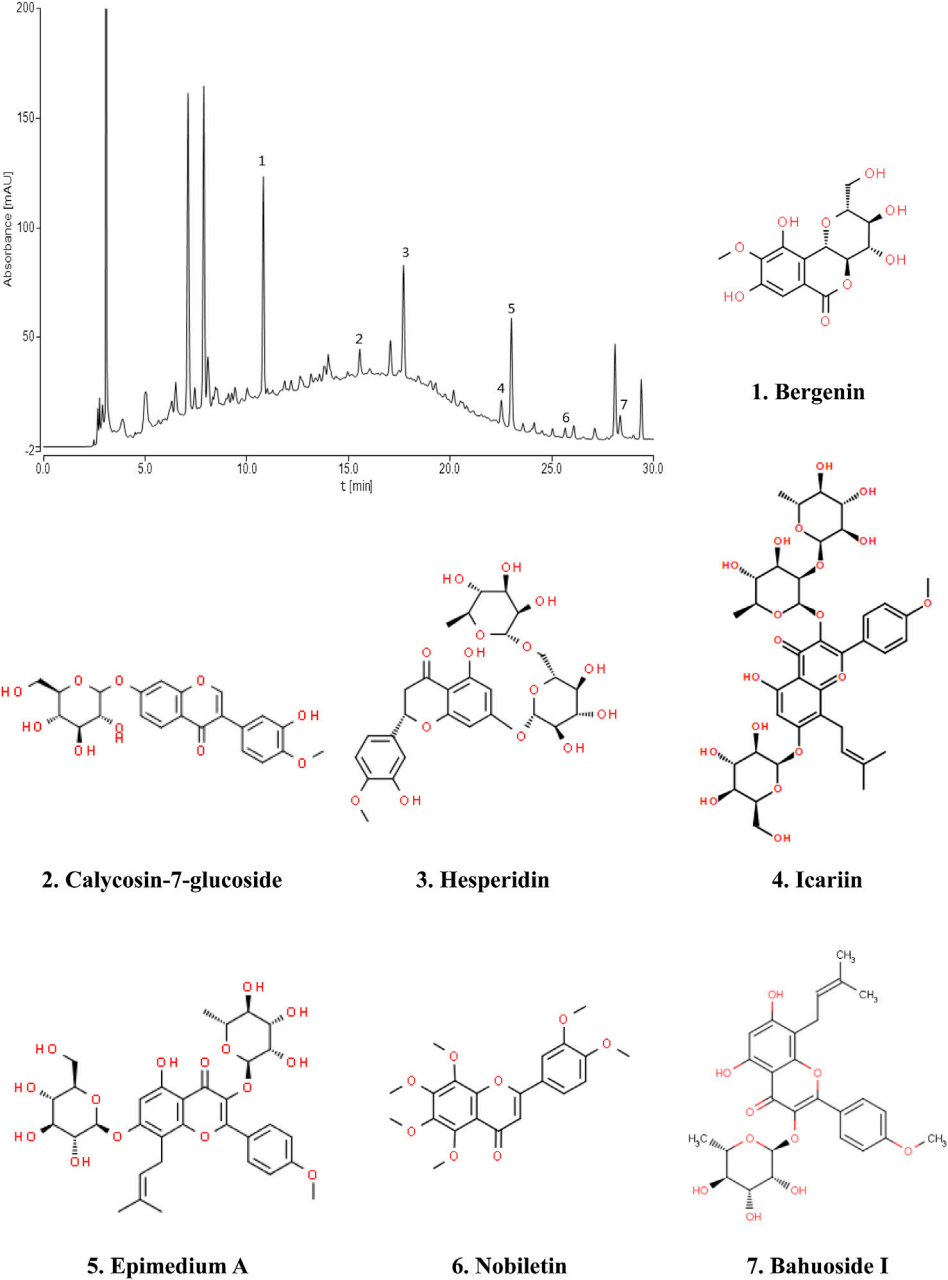
Chromatogram fingerprint of Bufei Jianpi formula (BJF). The 7 compounds were identified in BJF: 1, Bergenin; 2, Calycosin-7-glucoside; 3, Hesperidin; 4, Icariin; 5, Epimedium A; 6, Nobiletin; 7, Baohuoside Ⅰ.

Fingerprint of BJF The retention time values of the identified compounds were compared with that of the reference substances. A representative chromatogram of BJF was shown in Figure 1. 7 compounds including Bergenin (1.3456 mg/g), Calycosin-7-glucoside (0.5616 mg/g), Hesperidin (1.1755 mg/g), Epimedium A (0.6869 mg/g), Icariin (1.1195 mg/g), Nobiletin (0.0951 mg/g), BaohuosidⅠ (0.0806 mg/g) were identified and their structures had also been shown.

### BJF Attenuated the Severity of Respiratory and Skeletal Muscle Dysfunction in COPD Rats

**FIGURE 2:**
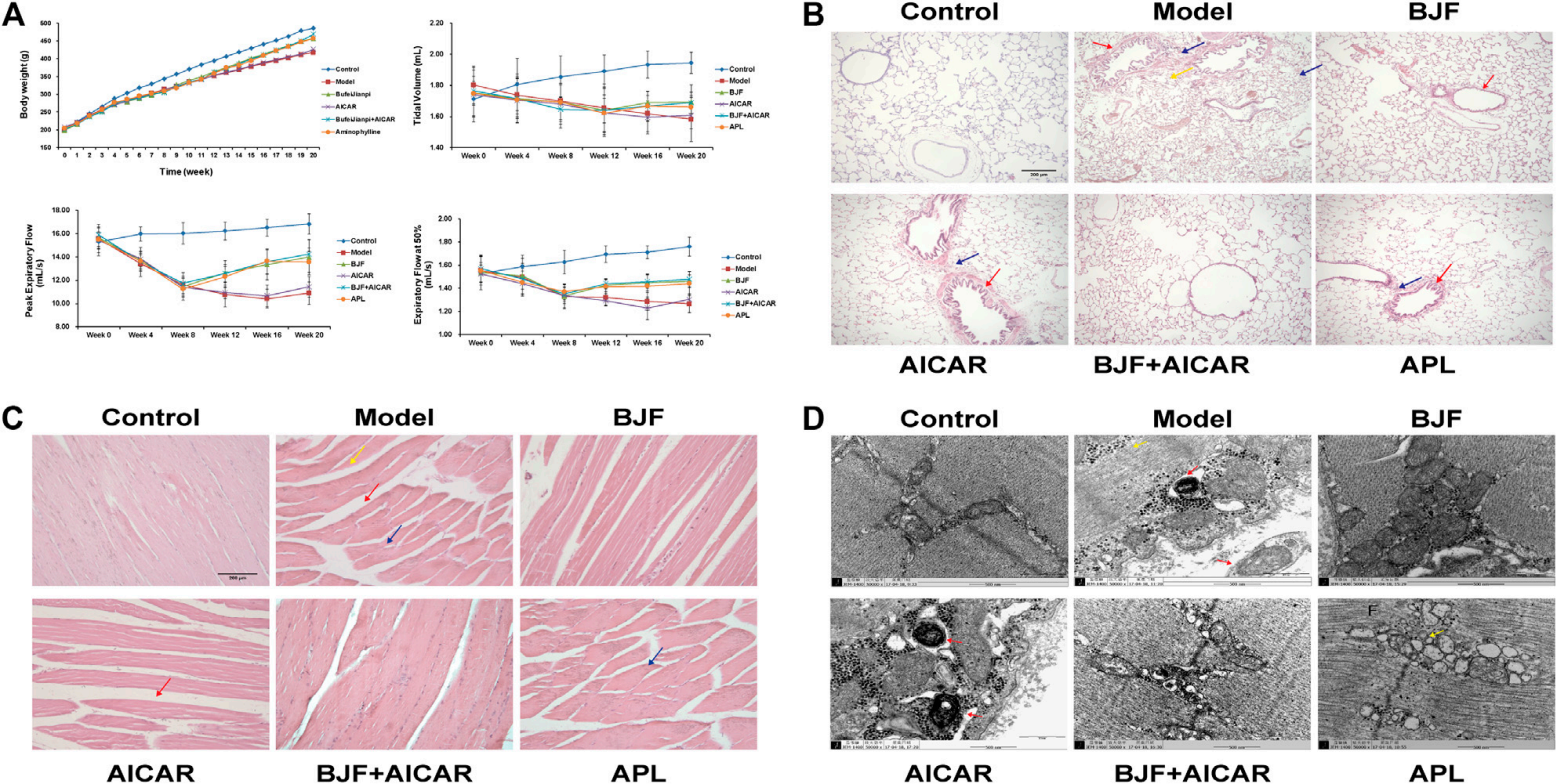
Effects of Bufei Jianpi formula (BJF) on COPD rats. **(A)** Body weight and pulmonary function [data are presented as mean ± SD (*n* = 6 per group)], **(B)** lung histomorphology, with red arrow indicating airway wall thickening, yellow arrow indicating pulmonary alveoli wall rupture, and blue arrow indicating inflammatory cell infiltration [hematoxylin and eosin (HE) staining, magnification ×100, scale bar = 100 μm], **(C)** skeletal muscle histomorphology, with red arrow indicating the increased intervals between muscle fibers, yellow arrow indicating muscle fiber atrophy and blue arrow indicating muscle fibers disarrangement (HE staining, magnification ×200, scale bar = 200 μm), and **(D)** skeletal muscle ultrastructure, with red arrow indicating mitochondrial autophagosomes fusing with lysosomes and yellow arrow indicating mitochondria complicated with vacuoles (magnification ×50,000). AICAR, 5-aminoimidazole-4-carboxamide ribonucleotide; APL, aminophylline.

To assess potential effects on skeletal and lung tissues, BJF was orally administered to COPD rats for 12 weeks using APL as a positive control. We found that BJF attenuated declines in body weight and respiratory function in COPD rats. As shown in Figure 2A, the body weight of rats in the model group increased slowly and was significantly lower than the control group at week 8. This decrease in body weight was markedly inhibited by treatment with BJF, APL, and AICAR, with no significant differences between the effects of these substances.

As shown in Tables 2–4, respiratory function analysis revealed that the PEF was significantly decreased in the model group at week 4 and that the VT and EF50 declined in the model group at week 8. Treatment with BJF, APL, and AICAR resulted in increased VT, PEF, and EF50.

**TABLE 2:** Tidal volume analysis.

Group	Week0	Week4	Week8	Week12	Week16	Week20
Control	1.71 ± 0.11	1.81 ± 0.17	1.86 ± 0.13	1.89 ± 0.11	1.93 ± 0.09	1.94 ± 0.07
COPD	1.80 ± 0.12	1.74 ± 0.14	1.70 ± 0.15^**^	1.65 ± 0.16^**^	1.62 ± 0.12^**^	1.58 ± 0.14^**^
BJF	1.75 ± 0.14	1.72 ± 0.13	1.69 ± 0.11	1.64 ± 0.15	1.69 ± 0.07	1.69 ± 0.11^##^
AICAR	1.74 ± 0.17	1.70 ± 0.14	1.68 ± 0.13	1.62 ± 0.15	1.59 ± 0.11^Δ^	1.61 ± 0.08^Δ^
BJF + AICAR	1.76 ± 0.09	1.72 ± 0.10	1.65 ± 0.08	1.64 ± 0.08	1.67 ± 0.10	1.69 ± 0.07^##^
APL	1.75 ± 0.15	1.71 ± 0.15	1.69 ± 0.17	1.62 ± 0.12	1.67 ± 0.09	1.66 ± 0.07^#^

Data are presented as mean ± SD (n = 6 per group). ^∗∗^ *P <* 0.01, ^∗^ *P <* 0.05 vs. control group;^##^ *P <* 0.01, ^#^ *P <* 0.05 vs. model group; ^ΔΔ^ *P* < 0.01, ^Δ^ *P* < 0.05 vs. BJF group; ^▲▲^ *P* < 0.01, ^▲^ *P* < 0.05 vs. 5-aminoimidazole-4-carboxamide ribonucleotide (AICAR) group; and ^□□^ *P* < 0.01, ^□^ *P* < 0.05 vs. BJF + AICAR group. APL, aminophylline.

**TABLE 3:** Peak expiratory flow analysis.

Group	Week0	Week4	Week8	Week12	Week16	Week20
Control	15.30 ± 1.18	15.97 ± 0.60	16.02 ± 0.93	16.22 ± 0.77	16.51 ± 0.72	16.83 ± 0.88
COPD	15.55 ± 1.22	13.40 ± 1.09^**^	11.62 ± 1.00^**^	10.75 ± 1.04^**^	10.42 ± 0.78^**^	10.89 ± 0.97^**^
BJF	15.63 ± 0.93	13.75 ± 0.58	11.43 ± 0.91	12.64 ± 1.04^##^	13.35 ± 1.33^##^	13.98 ± 1.50^##^
AICAR	15.44 ± 0.70	13.83 ± 0.98	11.39 ± 0.85	10.93 ± 0.49^ΔΔ^	10.66 ± 0.92^ΔΔ^	11.43 ± 0.88^ΔΔ^
BJF + AICAR	15.88 ± 0.60	13.59 ± 0.58	11.74 ± 0.92	12.60 ± 0.77^##^	13.57 ± 1.04^##▲▲^	14.23 ± 0.56^##▲▲^
APL	15.50 ± 0.59	13.64 ± 0.85	11.24 ± 0.94	12.29 ± 0.90^##^	13.63 ± 1.08^##▲▲^	13.57 ± 0.90^##^

Data are presented as mean ± SD (*n* = 6 per group). ^∗∗^ *P <* 0.01, ^∗^ *P <* 0.05 vs. control group;^##^ *P <* 0.01, ^#^ *P <* 0.05 vs. model group; ^ΔΔ^ *P* < 0.01, ^Δ^ *P* < 0.05 vs. BJF group; ^▲▲^ *P* < 0.01, ^▲^ *P* < 0.05 vs. 5-aminoimidazole-4-carboxamide ribonucleotide (AICAR) group; and ^□□^ *P* < 0.01, ^□^ *P* < 0.05 vs. BJF + AICAR group. APL, aminophylline.

**TABLE 4:** 50% tidal volume expiratory flow analysis.

Group	Week0	Week4	Week8	Week12	Week16	Week20
Control	1.53 ± 0.14	1.59 ± 0.10	1.63 ± 0.10	1.69 ± 0.07	1.71 ± 0.06	1.76 ± 0.08
COPD	1.55 ± 0.12	1.50 ± 0.10	1.33 ± 0.11^*^	1.32 ± 0.07^**^	1.29 ± 0.08^**^	1.27 ± 0.08^**^
BJF	1.54 ± 0.08	1.51 ± 0.15	1.33 ± 0.10	1.42 ± 0.05^##^	1.45 ± 0.07^##^	1.46 ± 0.06^##^
AICAR	1.53 ± 0.08	1.44 ± 0.11	1.34 ± 0.07	1.29 ± 0.04^ΔΔ^	1.23 ± 0.10^ΔΔ^	1.30 ± 0.05^ΔΔ^
BJF + AICAR	1.57 ± 0.12	1.48 ± 0.14	1.35 ± 0.06	1.44 ± 0.04^##▲▲^	1.46 ± 0.06^##▲▲^	1.48 ± 0.07^##▲▲^
APL	1.55 ± 0.12	1.45 ± 0.12	1.37 ± 0.07	1.41 ± 0.06^##▲▲^	1.42 ± 0.10^##▲▲^	1.44 ± 0.10^##▲▲^

Data are presented as mean ± SD (n = 6 per group). ^∗∗^ *P <* 0.01, ^∗^ *P <* 0.05 vs. control group;^##^ *P <* 0.01, ^#^ *P <* 0.05 vs. model group; ^ΔΔ^ *P* < 0.01, ^Δ^ *P* < 0.05 vs. BJF group; ^▲▲^ *P* < 0.01, ^▲^ *P* < 0.05 vs. 5-aminoimidazole-4-carboxamide ribonucleotide (AICAR) group; and ^□□^ *P* < 0.01, ^□^ *P* < 0.05 vs. BJF + AICAR group. APL, aminophylline.

We also found that BJF obviously reduced lung injury identified on histopathological examination. As shown in Figure 2B, the structure of the pulmonary alveoli and airways were fully intact in control rats, while marked airway wall thickening, alveolar wall rupture and inflammatory cell infiltration were observed in the lungs of COPD rats. These pulmonary histopathological impairments were significantly improved in rats treated with BJF, APL, and AICAR, with reductions in inflammatory cell infiltration, especially in the BJF and BJF + AICAR groups.

BJF also obviously improved morphological and ultrastructural changes in skeletal muscle of COPD rats. As shown in Figures 2C,D, marked morphological changes, including muscle fiber atrophy and disarrangement, increased intervals between muscle fibers, uneven myocyte cytoplasmic staining, and differently sized nuclei were observed in the skeletal muscle of model rats. These changes were improved after treatment with BJF, APL, and AICAR, particularly in the BJF and BJF + AICAR groups. While the model, APL, and AICAR groups showed marked ultrastructural changes, including an abundance of swollen mitochondria with increased number of vacuoles, a fracturecrest of the mitochondria, and increased mitophagy, BJF ameliorated these abnormal mitochondrial findings.

### BJF Ameliorated Mitochondrial Function in COPD Rats

**FIGURE 3:**
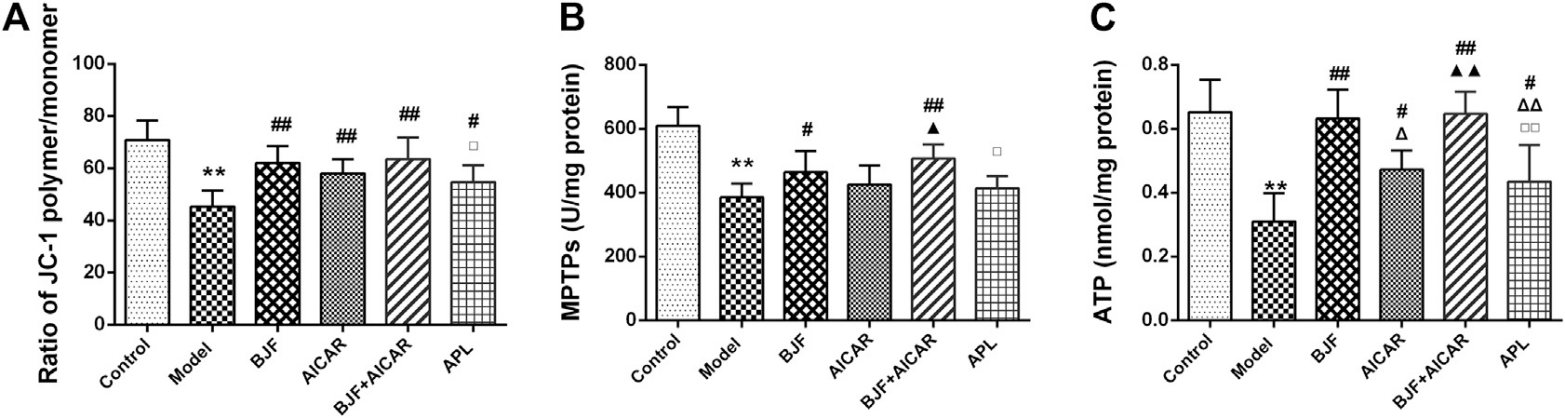
Effects of Bufei Jianpi formula (BJF) on mitochondrial function of COPD rats. **(A)** Mitochondrial membrane potential **(B)** mitochondrial permeability transition pore openings (MPTPs); and **(C)** adenosine triphosphate (ATP) levels. Data are presented as mean ± SD (*n* = 6 per group). ∗∗*p <* 0.01, ∗*p <* 0.05 vs. control group; ^##^ *p <* 0.01, ^#^ *p <* 0.05 vs. model group; ^ΔΔ^ *P* < 0.01, ^Δ^ *P* < 0.05 vs. BJF group; ^▲▲^ *p* < 0.01, ^▲^ *p* < 0.05 vs. 5-aminoimidazole-4-carboxamide ribonucleotide (AICAR) group; and ^□□^ *p* < 0.01, ^□^ *p* < 0.05 vs. BJF + AICAR group. APL, aminophylline.

To confirm the effect of BJF on mitochondrial function, we measured the levels of mitochondrial functional indicators. As shown in Figures 3A–C, MMPs, MPTP openings, and ATP levels in model rats were significantly lower than in control rats, with BJF administration significantly increasing MMPs, MPTP openings, and ATP levels. Meanwhile, AICAR and APL increased levels of MMPs and ATP but had no effect on MPTP openings.

BJF Promoted Expression of Mitochondrial Biogenesis Factors via Regulation of the AMPK Signaling Pathway in COPD Rats

**FIGURE 4:**
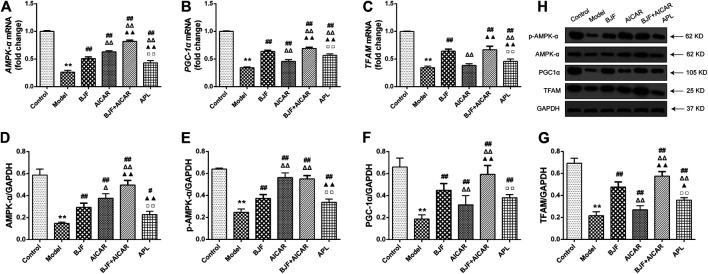
Effects of Bufei Jianpi formula (BJF) on expression of mitochondrial biogenesis factors in COPD rats. **(A–C)** Real-time polymerase chain reaction validation of adenosine monophosphate-activated protein kinase (*AMPK-α*), peroxisome proliferator-activated receptor gamma coactivator (*PGC-α*), and mitochondrial transcription factor A (*TFAM*) mRNA expression changes and **(D–H)** representative Western blot analysis of AMPK-α, *p*-AMPK-α, PGC-α, and TFAM protein expression levels. Data are shown as mean ± SD (n = 6 per group). ^∗∗^ *p <* 0.01, ^∗^ *p <* 0.05 vs. control group; ^##^ *p <* 0.01, ^#^ *p <* 0.05 vs. model group; ^ΔΔ^ *P* < 0.01, ^Δ^ *P* < 0.05 vs. BJF group; ^▲▲^ *p* < 0.01, ^▲^ *p* < 0.05 vs. 5-aminoimidazole-4-carboxamide ribonucleotide (AICAR) group; ^□□^ *p* < 0.01, ^□^ *p* < 0.05 vs. BJF + GAPDH, glyceraldehyde 3-phosphate dehydrogenase.

To explore the mitochondrial biogenesis-related mechanisms of BJF, we measured mRNA and protein levels of AMPK-α, PGC-α, and TFAM in skeletal muscle tissues of COPD rats. As shown in Figures 4A–C, mRNA levels of *AMPK-α*, *PGC-α*, and *TFAM* were markedly decreased in model rats. While treatment with BJF and APL significantly promoted expression of *AMPK-α*, *PGC-α*, and *TFAM* mRNA, AICAR upregulated levels of *AMPK-α* and *PGC-α* but not TFAM. As shown in Figures 4D–H, protein expression of AMPK-α, *p*-AMPK-α, PGC-α, and TFAM were lowered in model rats compared with control rats. BJF, AICAR, and APL treatment resulted in dramatic increases in AMPK-α, *p*-AMPK-α, PGC-α, and TFAM protein expression.

BJF Downregulated Expression of Mitophagic Factors via Regulation of the AMPK Signaling Pathway in COPD Rats

**FIGURE 5:**
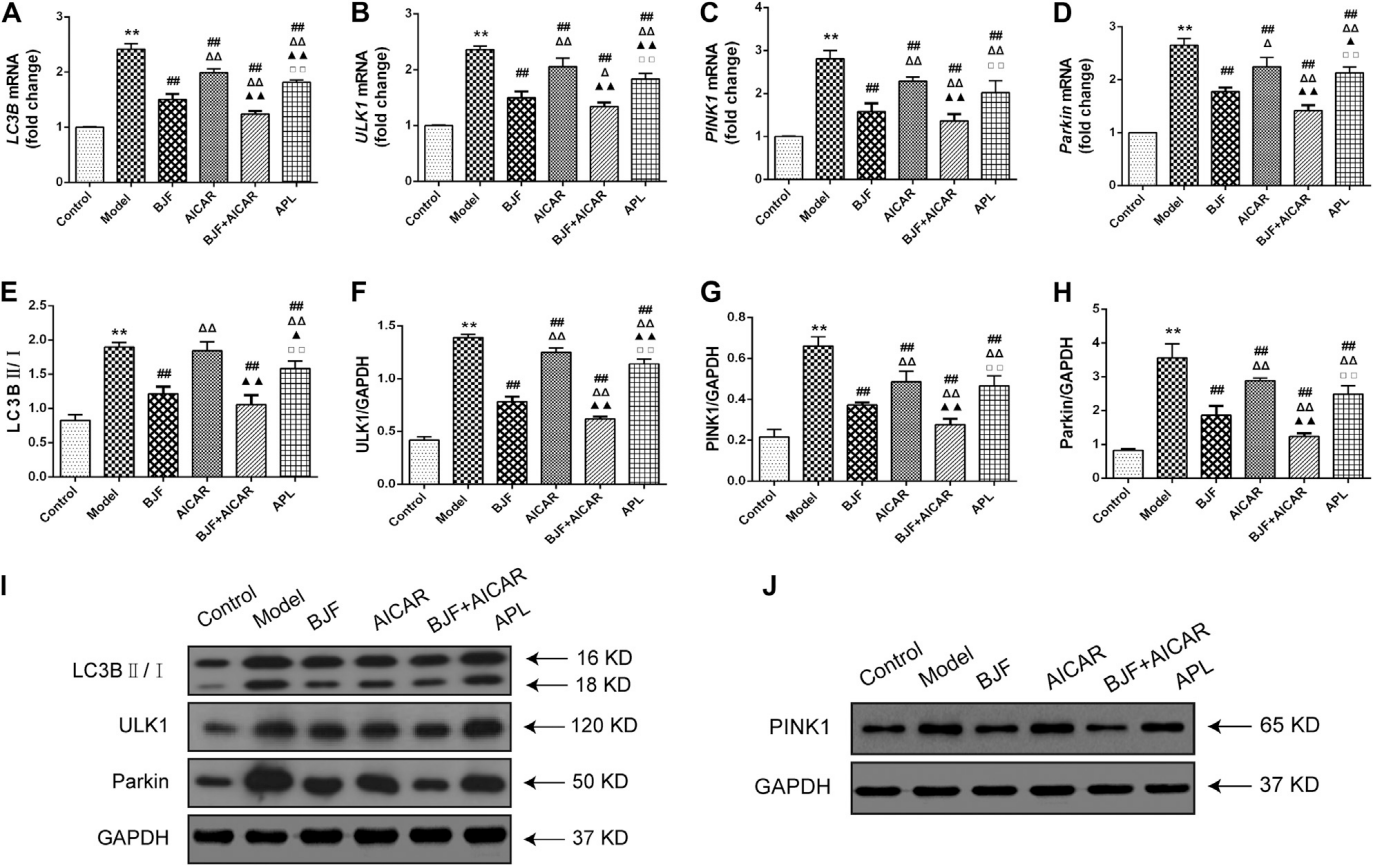
Effects of Bufei Jianpi formula (BJF) on expression of mitophagic factors in COPD rats. **(A–D)** Real-time polymerase chain reaction validation of light chain 3B (*LC3B*), UNC-51-like kinase (*ULK1*), PTEN-induced putative kinase 1 (*PINK1*), and P*arkin* mRNA expression levels. **(E–J)** Representative Western blot analysis of LC3B, ULK1, PINK1, and Parkin protein expression. Data are presented as mean ± SD (*n* = 6 per group). ∗∗*p <* 0.01, ∗*p <* 0.05 vs. control group; ^##^ *p <* 0.01, ^#^ *p <* 0.05 vs. model group; ^ΔΔ^ *P* < 0.01, ^Δ^ *P* < 0.05 vs. BJF group; ^▲▲^ *p* < 0.01, ^▲^ *p* < 0.05 vs. 5-aminoimidazole-4-carboxamide ribonucleotide (AICAR) group; ^□□^ *p* < 0.01, ^□^ *p* < 0.05 vs. BJF + AICAR group. GAPDH, glyceraldehyde 3-phosphate dehydrogenase.

To identify the mitophagy-related mechanisms of BJF, we measured mRNA and protein levels of LC3B, ULK1, PINK1, and Parkin in skeletal muscle tissues of COPD rats. As shown in Figures 5A–D, we found that *LC3B*, *ULK1*, *PINK1*, and *Parkin* mRNA levels were significantly upregulated in COPD rats in comparison to control rats. While treatment with BJF, AICAR, and APL downregulated expression of *LC3B*, *ULK1*, *PINK1*, and *Parkin* mRNA, AICAR upregulated levels of all the above-mentioned indicators. Additionally, protein expression of LC3B, ULK1, PINK1, and Parkin were markedly decreased in COPD rats compared with control rats. While BJF and APL treatment upregulated protein expression of LC3B, ULK1, PINK1, and Parkin, AICAR showed no effect on the level of LC3B (Figures 5E–J).

### BJF Improved CSE-Induced Mitochondrial Injury in L6 Cells

**FIGURE 6:**
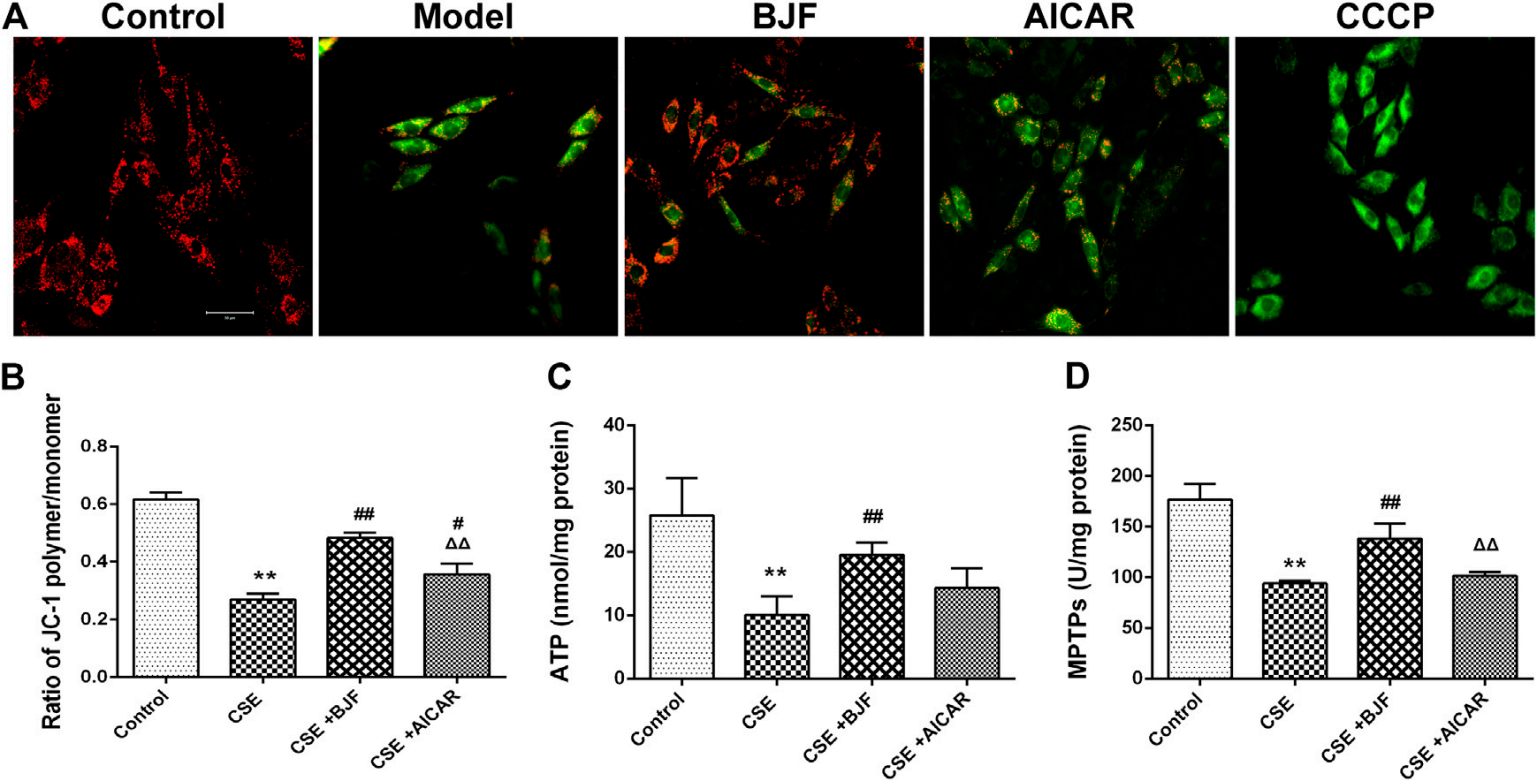
Bufei Jianpi formula (BJF) improved cigarette smoke extract (CSE)-induced mitochondrial injury in L6 cells. **(A)** Effect of BJF on mitochondrial membrane potential. Red fluorescence represents the mitochondrial aggregate form of JC-1, indicating an intact mitochondrial membrane potential. Green fluorescence represents the monomeric form of JC-1, indicating dissipation of ΔΨm (confocal laser scanning microscope, magnification ×200, scale bar = 50 μm), **(B)** mitochondrial membrane potential, **(C)** ATP, and **(D)** mitochondrial permeability transition pore (MPTP) openings. Data are presented as mean ± SD. ∗∗*p <* 0.01, ∗*p <* 0.05 vs. control group; ^##^ *p <* 0.01, ^#^ *p <* 0.05 vs. model group; ^ΔΔ^ *P* < 0.01, ^Δ^ *P* < 0.05 vs. BJF group. AICAR, aminoimidazole-4-carboxamide ribonucleotide; CCCP, carbonyl cyanide *m*-chlorophenyl hydrazone.

To explore the effects of BJF treatment on mitochondrial membrane potentials, a JC-1 fluorescent probe was measured in rat L6 cells under CSE conditions. The ratio of JC-1 red to green fluorescence was significantly decreased in CSE-exposed L6 cells; however, treatment with BJF and AICAR significantly increased MMPs in L6 cells (Figures 6A,B). As shown in Figures 6C,D, BJF also markedly improved opening of MPTPs and ATP levels in CSE-exposed L6 cells.

### BJF Restored Mitochondrial Respiration in CSE-Exposed L6 Cells

**FIGURE 7:**
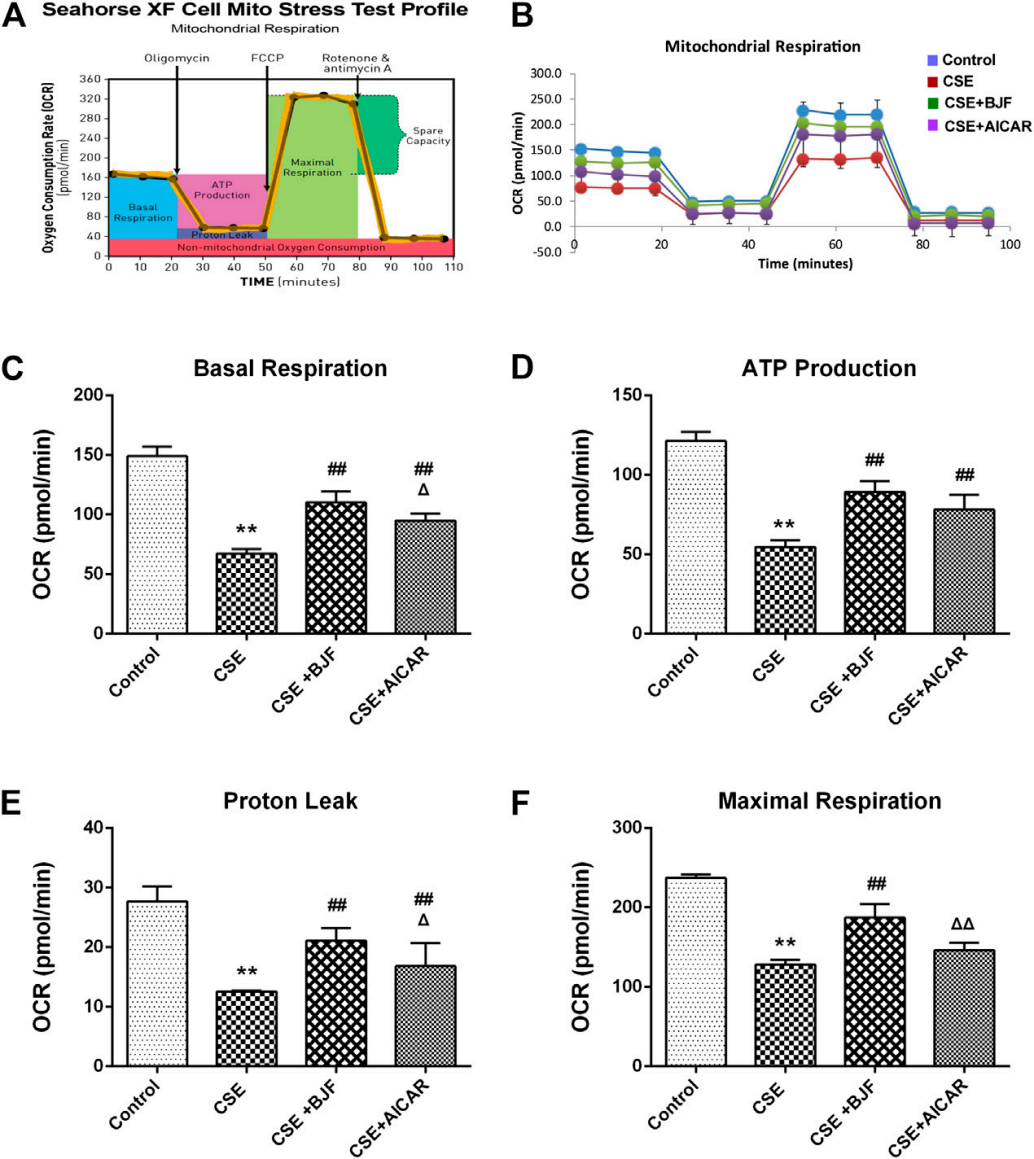
Bufei Jianpi formula (BJF) restored mitochondrial respiration in cigarette smoke extract (CSE)-exposed L6 cells. **(A)** Schematic representation of the mitochondrial functional assay, and **(B)** mitochondrial respiration under CSE conditions. L6 cells were treated with 10% CSE, 10% CSE +10% BJF-containing serum, or 10% CSE + 5-aminoimidazole-4-carboxamide ribonucleotide (AICAR) for 24 h. The cells were then switched to unbuffered Dulbecco’s modified Eagle’s medium supplemented with pyruvate, followed by real-time analysis of oxygen consumption rate (OCR). Analysis of **(C)** basal respiration, **(D)** adenosine triphosphate (ATP) production, **(E)** proton leak, and **(F)** maximal respiration. Data are presented as mean ± SD. ∗∗*p <* 0.01, ∗*p <* 0.05 vs. control group; ^##^ *p <* 0.01, ^#^ *p <* 0.05 vs. model group; ^ΔΔ^ *P* < 0.01, ^Δ^ *P* < 0.05 vs. BJF group.

To determine the effects of BJF on mitochondrial respiration, L6 cells were treated with a 10% CSE, 10% CSE +10% BJF-containing serum, or a 10% CSE + AICAR for 24 h, and OCRs were measured. As demonstrated in Figures 7B–F **,** significant reductions in basal respiration, ATP production, proton leak, and maximal respiratory capacity were observed in L6 cells after exposure to CSE. BJF treatment markedly reversed these CSE-induced changes.

### BJF Regulated Expression of Mitochondrial Biogenesis Factor in CSE-Exposed L6 Cells

**FIGURE 8:**
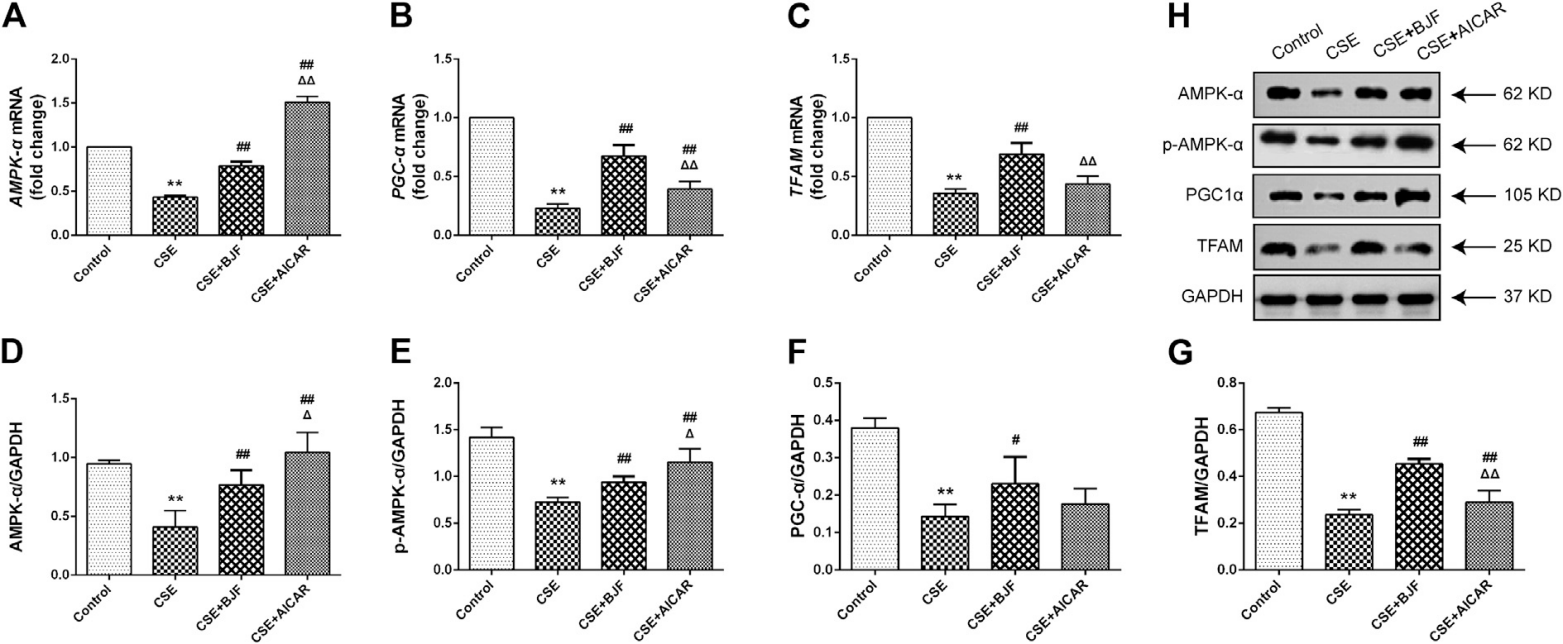
Bufei Jianpi formula (BJF) regulated expression of mitochondrial biogenesis factors in cigarette smoke extract (CSE)-exposed L6 cells. **(A–C)** Real-time polymerase chain reaction validation of adenosine monophosphate-activated protein kinase (*AMPK-α*), peroxisome proliferator-activated receptor gamma coactivator (*PGC-α*), and mitochondrial transcription factor A (*TFAM*) mRNA expression levels and **(D-H)** Representative Western blot analysis of AMPK-α, *p*-AMPK-α, PGC-α, and TFAM protein expression levels. Data are presented as mean ± SD. ∗∗*p <* 0.01, ∗*p <* 0.05 vs. control group; ^##^ *p <* 0.01, ^#^ *p <* 0.05 vs. model group; ^ΔΔ^ *P* < 0.01, ^Δ^ *P* < 0.05 vs. BJF group. GAPDH, glyceraldehyde 3-phosphate dehydrogenase.

To explore the effects of BJF on mitochondrial biogenesis, mRNA and protein levels of AMPK-α, PGC-α, and TFAM were measured in L6 cells exposed to CSE. As shown in Figures 8A–C, mRNA expression levels of *AMPK-α*, *PGC-α*, and *TFAM* were markedly lower in L6 cells exposed to CSE than in control cells, and BJF administration markedly reversed these changes. Meanwhile, AICAR upregulated levels of *AMPK-α* and *PGC-α* but had no effect on levels of *TFAM*. As shown in Figures 8D–H, protein expression of AMPK-α, *p*-AMPK-α, PGC-α, and TFAM were lowered in L6 cells exposed to CSE compared with control cells. BJF treatment resulted in dramatic increases in these changes,while AICAR upregulated levels of AMPK-α, *p*-AMPK-α and TFAM.

### BJF Downregulated Expression of Mitophagic Factor in CSE-Exposed L6 Cells

**FIGURE 9:**
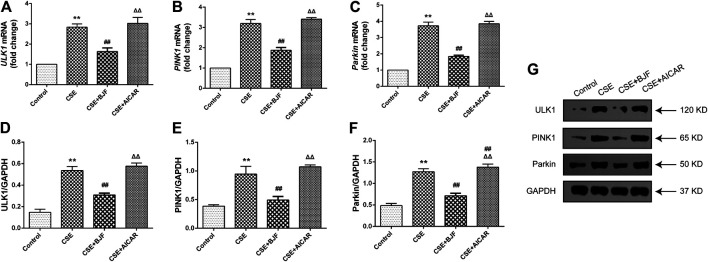
Bufei Jianpi formula (BJF) downregulated expression of mitochondrial autophagic factors in cigarette smoke extract (CSE)-exposed L6 cells. **(A–C)** Real-time polymerase chain reaction validation of UNC-51-like kinase (*ULK1*), PTEN-induced putative kinase 1 (*PINK1*), and *Parkin* mRNA expression levels and **(D–G)** Representative Western blot analysis of ULK1, PINK1, and Parkin protein expression levels. Data are presented as mean ± SD. ∗∗*p <* 0.01, ∗*p <* 0.05 vs. control group; ^##^ *p <* 0.01, ^#^ *p <* 0.05 vs. model group; ^ΔΔ^ *P* < 0.01, ^Δ^ *P* < 0.05 vs. BJF group. GAPDH, glyceraldehyde 3-phosphate dehydrogenase.

We measured mRNA and protein levels of ULK1, PINK1, and Parkin in L6 cells under CSE conditions. As shown in Figures 9A–G, *ULK1*, *PINK1*, and *Parkin* mRNA levels were significantly upregulated in CSE-induced L6 cells in comparison to control cells. Meanwhile, BJF treatment downregulated *ULK1*, *PINK1*, and *Parkin* mRNA and protein expression levels. Similarly, AICAR increased Parkin protein expression.

### BJF Regulated the AMPK Signaling Pathway in CSE-Exposed L6 Cells

**FIGURE 10:**
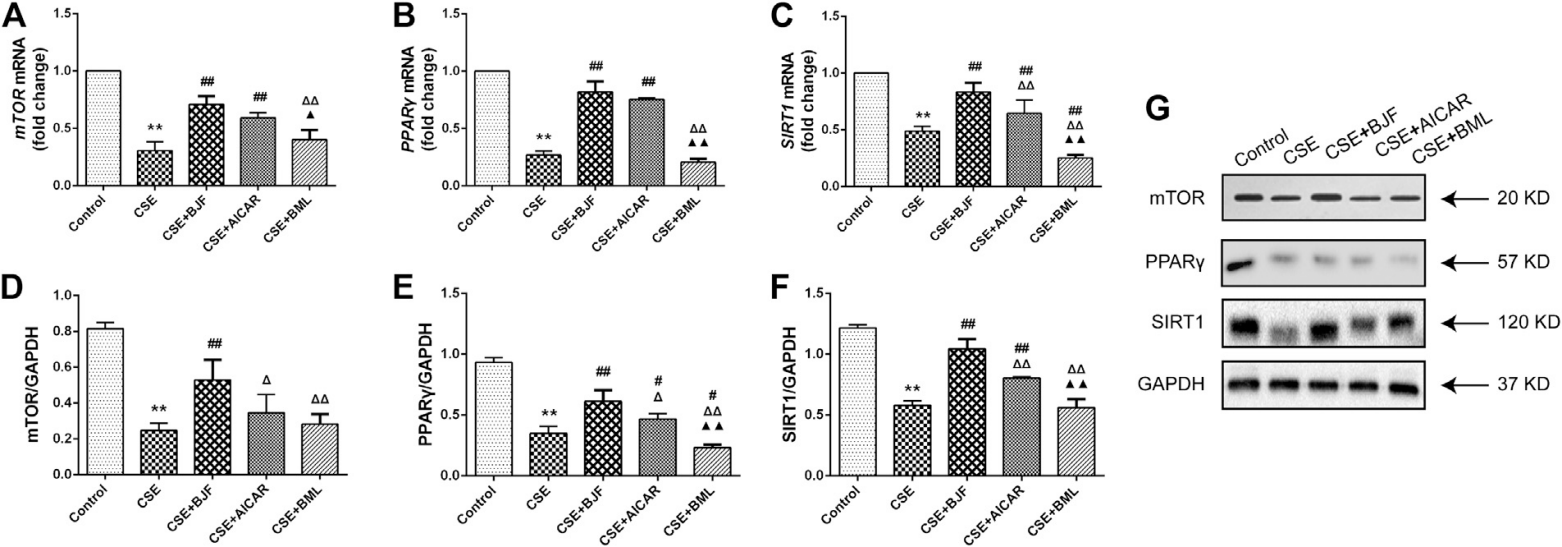
Bufei Jianpi formula (BJF) regulated the adenosine monophosphate-activated protein kinase (AMPK) signaling pathway in cigarette smoke extract (CSE)-exposed L6 cells. **(A–C)** Real-time polymerase chain reaction validation of mammalian target of rapamycin (*mTOR*), *PPARγ*, and *SIRT1* mRNA expression levels and **(D–G)** Representative Western blot analysis of mTOR, peroxisome proliferator-activated receptor (PPAR)-γ, and sirtuin 1 (SIRT1) protein expression levels. Data are presented as mean ± SD. ∗∗*p <* 0.01, ∗*p <* 0.05 vs. control group; ^##^ *p <* 0.01, ^#^ *p <* 0.05 vs. model group; ^ΔΔ^ *P* < 0.01, ^Δ^ *P* < 0.05 vs. BJF group; ^▲▲^ *p* < 0.01, ^▲^ *p* < 0.05 vs. AICAR group. GAPDH, glyceraldehyde 3-phosphate dehydrogenase.

To further explore the effects of BJF treatment on the AMPK signaling pathway, mRNA and protein levels of factors downstream of AMPK, including mTOR, PPARγ, and SIRT1 were analyzed. As shown in Figures 10A–G, mRNA levels of *mTOR*, *PPARγ*, and *SIRT1* were significantly decreased after administration of CSE for 24 h, and treatment with BJF and AICAR inhibited these changes. Similarly, BJF increased protein expression of mTOR, PPARγ, and SIRT1, while AICAR upregulated levels of PPARγ and SIRT1 but had no effect on mTOR levels. BML increased mRNA expression of *PPARγ* , SIRT1and protein expression of PPARγ.

## Discussion

**FIGURE 11:**
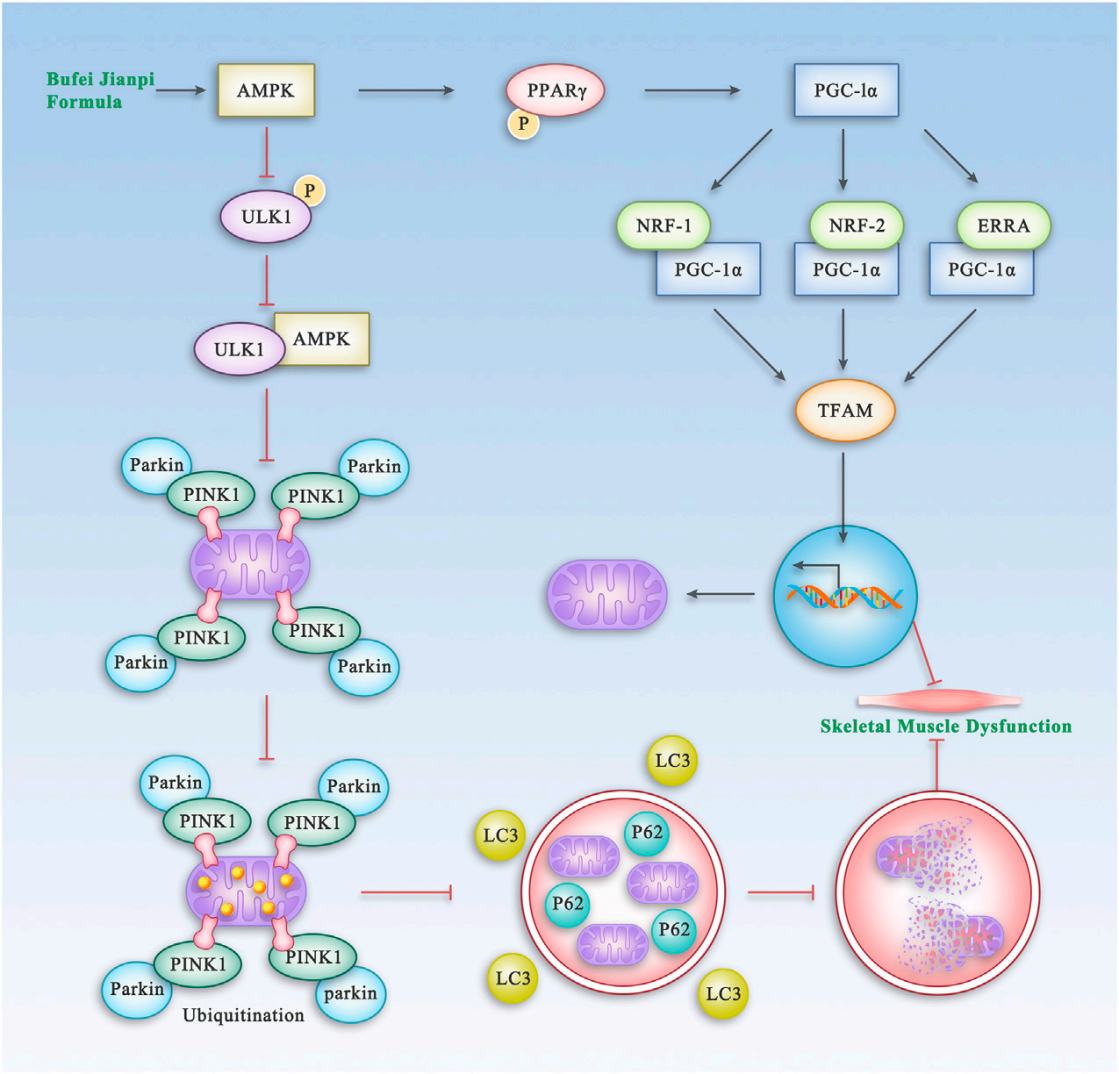
The Proposed Pathway of BJF for the Treatment of Skeletal Muscle Dysfunction in COPD. BJF improves skeletal muscle and mitochondrial function in COPD rats and L6 cells by promoting mitochondrial biogenesis and suppressing mitophagy via the AMPK pathway.

TCM has been widely used for treatment of COPD for thousands of years. BJF, a traditional Chinese herbal formula for lung-spleen qi deficiency syndrome, has been shown to have reproducible efficacy for treatment of COPD in previous clinical and experimental studies (Li et al., 2012a; Dong et al., 2015; Mao et al., 2019). In this study, we showed that BJF significantly improved SMD in rats with COPD via regulation of the AMPK pathway which is shown in Figure 11. It also improved mitochondrial function and decreased mitophagy in model rats and a myocyte cell line exposed to cigarette smoke extract.

Mitochondria, an important and essential organelle in the skeletal muscle, is involved in metabolic regulation and ATP production, which are two key elements of muscle contractibility and plasticity (Abrigo, 2019). Many evidence has accumulated to support the notion that mitochondrial dysfunction contribute to the poor endurance capacity and atrophy that characterize locomotor muscle dysfunction in patients with COPD (Allaire, 2004). There are several potential therapeutic agents targeted at reducing mitochondrial impairment in skeletal muscle of patients with COPD, including polyunsaturated fatty acids (PUFA), exercise rehabilitation, and mitochondria-targeting antioxidant therapies (Taivassalo and Hussain, 2016). TCM has also been shown to have clinically beneficial effects on mitochondrial impairment. For example, Astragalus polysaccharide has been shown to improve mitochondrial dysfunction via inhibiting the opening of a mitochondrial permeability transition pore, improving synergistic interactions between mitophagy and mitochondrial fusion. (Huang et al., 2016; Sun et al., 2014.). Our previous study also showed that BJF reversed the reductions in mitochondrial density observed in COPD rats and improved skeletal muscle tension and tolerance (Dong et al., 2015). In the present study, we demonstrated that BJF treatment elevated indicators of effective mitochondrial functioning, including ATP, MMP, and MPTP openings in COPD rats, as well as restored levels of mitochondrial respiration in CSE-exposed L6 cells.

Mitochondria use oxidative phosphorylation to synthesize ATP, which provides 90% of the energy required for all cellular activities (Zhang L. et al., 2017). Thus, damage to the mitochondria reduces ATP synthesis; this not only directly leads to mitochondrial swelling, disruption of cristae, and uncoupling of oxidative phosphorylation but also exacerbates ion metabolism disorders (Formentini et al., 2017). Repeated vicious cycles ultimately lead to myocyte damage and cause impairment. Several studies have found that the skeletal muscle cells of patients with COPD hold features of mitochondrial dysfunction, such as increased synthesis of ROS and decreased ATP production (Puente-Maestu et al., 2009). A normal mitochondrial membrane potential is a prerequisite for maintaining oxidative phosphorylation and ATP synthesis, to ensure normal mitochondrial activity. The MPTP is located between the outer and inner mitochondrial membranes and is a nonselective complex pore composed of several proteins. The MPTP cyclical opening maintains the calcium ion flow, pH, and charge equilibrium in the mitochondrial matrix, ensuring a stable membrane potential while maintaining mitochondrial homeostasis. Pathological or other factors can stimulate the MPTP and induce its excessive opening, which results in the influx of a large number of protons from the inner membrane to the matrix and, hence, in the loss of mitochondrial membrane potential, uncoupling of oxidative phosphorylation, and ATP synthesis failure. In addition, aberrant opening of the MPTP may allow solute molecules to freely pass through the mitochondrial matrix and trigger significant mitochondrial swelling. Ultimately, mitochondrial swelling can lead to the disruption of the mitochondrial outer membrane and the release of cytochrome C and other pro-apoptotic factors, thereby activating apoptosis or necrosis pathways (Bernardi et al., 2015; Ong et al., 2015; Teixeira et al., 2018). One study found that MPTP opening is aberrant in the skeletal muscles of COPD patients (Puente-Maestu et al., 2009). In contrast, another study reported that COPD patients were less prone to aberrant MPTP opening in the skeletal muscles compared with healthy individuals (Picard et al., 2008). To date, relatively few studies have explored the MMP and MPTP opening in the skeletal muscles of COPD patients; therefore, further research on these factors is warranted. This study showed that ATP synthesis, MMP levels, and MPTP opening were significantly increased in BJF and BJF+AICAR group. These results suggest that “the spleen is the source of qi and blood” and is closely associated with mitochondrial function. In addition, BJF can significantly improve mitochondrial function, thereby ameliorating skeletal muscle atrophy.

The mitochondria are essential for cellular aerobic respiration, partaking in stages 2 and 3 (i.e., Krebs cycle and oxidative phosphorylation). Our study measured the respiratory characteristics of mitochondria under cellular baseline and stress conditions. there is evidence indicated that mitochondrial aerobic respiration was inhibited in L6 skeletal muscle cells exposed to high-dose fructose, with significantly reduced oxygen consumption rate, basal and maximal respiration, and ATP synthesis (Jaiswal et al., 2015). Nevertheless, another study found that resveratrol could partially overcome dexamethasone-induced impaired mitochondrial respiration in C2C12 myoblasts, thereby preventing mitochondrial dysfunction. The results of this study showed that treatment with CSE could decrease basal mitochondrial oxygen consumption rate, ATP synthesis, and proton leakage in L6 cells (Liu et al., 2016). Taken together, these results suggest that CSE-induced mitochondrial dysfunction and BJF can significantly increase aerobic respiration in mitochondria.

Different factors related to mitochondrial dysfunction, including abnormalities in mitochondrial biogenesis and mitophagy, may contribute to the loss of skeletal muscle strength that leads to functional declines in patients with COPD (Wiegman et al., 2015). AMPK, a vital regulator of bioenergetic metabolism, is critical for mitochondrial biogenesis and mitophagy in muscle cells (Zhang et al., 2018). AMPK activates TFAM via regulation of PGC-1α and NRF, which contributes to mitochondrial biogenesis when activated by hypoxia and oxidative stress. PGC-1α has been shown to play a central role in enhancing mitochondrial biogenesis (Islam et al., 2018). TFAM is another key molecule in the regulation of mtDNA copy numbers and mitochondrial function (Kang et al., 2018). Emerging evidence indicates that levels of PGC-1α and TFAM are decreased in COPD rats (Remels et al., 2007; Lu et al., 2017; Zhang M. et al., 2017). Meanwhile, other studies have demonstrated that protein expression of PGC-1α is significantly lower in patients with COPD due to decreased numbers of type I fibers (van den Borst et al., 2013; Puente-Maestu et al., 2013). These studies suggest that these factors may serve important regulatory functions in COPD-related mitochondrial dysfunction. In the present study, BJF was shown to effectively upregulate mitochondria biogenesis by increasing expression levels of AMPK-α, PGC-1α, and TFAM.

The PINK1/Parkin pathway is one of the main molecular mechanisms mediating the mitophagic process. A previous study showed that the number of autophagosomes was clearly increased in skeletal muscles of patients with COPD, which was associated with functional impairments in lungs and muscular atrophy. Increased expressions of LC3BII/I and *p*-ULK have also been observed in COPD patients (Kneppers et al., 2017), indicating abnormalities in autophagy. In the present study, we found that the levels of ULK1, PINK1, Parkin, and LC3B were significantly increased in the skeletal muscle of COPD rats, while treatment with BJF or APL could inhibit mitophagy to different degrees. MTOR is a 289-kDa serine/threonine protein involved in multiple cellular processes, including autophagy (Maiese, 2016). AMPK plays an important role in the signaling cascade of mTOR by inhibiting mTORC1 activity, which results in induction of autophagy (Duan et al., 2016). SIRT1 is a downstream target of AMPK and is therefore downregulated when *p*-AMPK is downregulated (Qi et al., 2014). In the present study, we found that BJF upregulated expression of mTOR, PPARγ, and SIRT1 via regulation of the AMPK signaling pathway in CSE-exposed L6 cells. Therefore, these results suggest that BJF can improve mitochondrial dysfunction by suppressing mitophagy and enhancing mitochondrial biogenesis via regulation of the AMPK signaling pathway.

Nevertheless, further study is needed to figure out which chemical components in BJF are responsible for suppressing mitophagy and enhancing mitochondrial biogenesis, maybe other mechanism involve in this process.

## Conclusion

In conclusion, this study demonstrated that BJF can improve the SMD observed in COPD both *in vitro* and *in vivo* by promotion of mitochondrial biogenesis and by suppression of mitophagy via regulation of the AMPK pathway. The results of this study indicate potential mechanisms for future therapeutic agents and provide a basis for further study of SMD in COPD patients.

## Data Availability Statement

The raw data supporting the conclusions of this article will be made available by the authors, without undue reservation, to any qualified researcher.

## Ethics Statement

The animal study was reviewed and approved by the Experimental Animal Care and Ethics Committee of the First Affiliated Hospital of Henan University of Traditional Chinese Medicine.

## Author Contributions

JM performed the research, analyzed the data, and wrote the manuscript. YL, YT, and SL designed and conceptualized the research and revised the manuscript. QB, JL, YH, LZ, and HJ contributed to animal experiments and cell culture. SF and XL contributed to the preparation of BJF. All authors read and approved the final manuscript.

## Funding

This work was supported by the General Program of National Natural Science Foundation of China (81573947 and 81904116) and the Scientific Research Fund for doctors of Henan University of Chinese Medicine (RSBSJJ2018-03).

## Conflict of Interest

The authors declare that the research was conducted in the absence of any commercial or financial relationships that could be construed as a potential conflict of interest.

## Glossary

AICAR: 5-aminoimidazole-4-carboxamide ribonucleotide
AMPK: adenosine monophosphate-activated protein kinase
APL: aminophylline
ATP: adenosine triphosphate
BCA: bicinchoninic acid
BFJ: Bufei Jianpi formula
CCCP: carbonyl cyanide *m*-chlorophenyl hydrazone
COPD: chronic obstructive pulmonary disease
CSE: cigarette smoke extract
DMEM: Dulbecco’s modified Eagle’s medium
EF-50: mid-tidal expiratory flow
FBS: fetal bovine serum
FCCP: carbonyl cyanide 4-(trifluoromethoxy) phenylhydrazone
Fis-1: mitochondrial fission-1 protein
GAPDH: glyceraldehyde 3-phosphate dehydrogenase
HE: hematoxylin and eosin
LC3B: light chain 3B
MMP: mitochondrial membrane potential
MPTP: mitochondrial permeability transition pores;
mTOR: mammalian target of rapamycin
NRF: nuclear respiratory factor
OCR: oxygen combustion rate
PEF: peak expiratory flow
PGC-1α: peroxisome proliferator-activated receptor gamma coactivator
PINK-1: PTEN-induced putative kinase 1
PPAR: peroxisome proliferator-activated receptor
PVDF: polyvinylidene difluoride
ROS: reactive oxygen species
SD: Sprague–Dawley
SDS-PAGE: sodium dodecyl sulfate polyacrylamide gel electrophoresis
SIRT1: (sirtuin 1)
SMD: skeletal muscle dysfunction
TCM: traditional Chinese medicine
TFAM: mitochondrial transcription factor A
TV: tidal volume
ULK-1: UNC-51-like kinase
